# Lymph node retrieval in colorectal cancer: determining factors and prognostic significance

**DOI:** 10.1007/s00384-017-2778-8

**Published:** 2017-02-16

**Authors:** Johannes Betge, Lars Harbaum, Marion J. Pollheimer, Richard A. Lindtner, Peter Kornprat, Matthias P. Ebert, Cord Langner

**Affiliations:** 1 Department of Medicine II, University Hospital Mannheim, Medical Faculty Mannheim, Heidelberg University, Mannheim, Germany; 2 Department of Medicine II, University Medical Center Hamburg-Eppendorf, Hamburg, Germany; 3 Institute of Pathology, Medical University of Graz, Auenbruggerplatz 25, 8036 Graz, Austria; 4 Department of Surgery, Medical University of Innsbruck, Innsbruck, Austria; 5 Department of Surgery, Division of General Surgery, Medical University of Graz, Graz, Austria

**Keywords:** Lymph node, Prognostic factor, Colon cancer, Rectum cancer, Multivariate analysis

## Abstract

**Purpose:**

The study aimed to analyze clinicopathological factors that determine the extent of lymph node retrieval and to evaluate its prognostic impact in patients with colorectal cancer (CRC).

**Methods:**

The number of retrieved lymph nodes was analyzed in 381 CRC specimens. Lymph node count was related to different clinicopathological variables by binary logistic regression. Progression-free survival (PFS) and cancer-specific survival (CSS) were determined using the Kaplan-Meier method and Cox regression models.

**Results:**

The median number of retrieved lymph nodes was 20 (mean 21 ± 10, range 1–65) in right-sided, 13 (16 ± 10, 1–66) in left-sided, and 15 (18 ± 11, 3–64) in rectal tumors. The number of retrieved lymph nodes was independently associated with T-classification (*p* < 0.001), N-classification (*p* = 0.014), and tumor size *(p* = 0.005) as well as right-sided tumor location (*p* = 0.012). There was no association with age, sex, tumor grade, mismatch-repair status, and lymph or blood vessel invasion. The longer the surgical specimen, the higher were the numbers of retrieved and positive lymph nodes (*p* < 0.001, respectively). In patients with locally advanced (T3/T4) tumors (*n* = 283), analysis of more than 12 lymph nodes was independently associated with PFS (HR = 0.63, *p* = 0.025) and CSS (HR = 0.54, *p* = 0.004). In the subset of T3/T4 N0 patients (*n* = 130), analysis of more than 12 lymph nodes similarly proved to be an independent predictor of outcome (PFS, HR = 0.48, *p =* 0.046; OS, HR = 0.41, *p* = 0.026).

**Conclusion:**

The number of retrieved lymph nodes is associated with higher tumor stage, tumor size, and right-sided location. Low lymph node count indicates adverse outcome in patients with locally advanced (T3/T4) disease.

## Introduction

Colorectal cancer (CRC) is globally the third most commonly diagnosed cancer in males and the second most common in females [1]. It is thus a major contributor to cancer morbidity and mortality worldwide.

In CRC, the involvement of lymph nodes by tumor cells represents a major step to systemic tumor spread and is therefore a strong indicator of adverse prognosis [2]. Lymph node involvement is a determining variable of the AJCC/UICC TNM system, which is currently the most relevant prognostic classification and used as basis for therapeutic decisions [3, 4]. Specifically, patients without evidence of distant metastasis undergoing resection for primary colon cancer may receive adjuvant chemotherapy mainly based upon the identification of metastatic lymph nodes [5].

Adequate lymphadenectomy and sufficient lymph node retrieval from the resected specimen are crucial to ensure accuracy in staging, especially to prevent under-diagnosis of lymph node involvement by tumor cells [6, 7]. Additionally, a higher number of sampled lymph nodes has emerged as independent prognostic factor for improved survival in several previous studies, especially in stage II CRC [6–11]. Hence, adjuvant chemotherapy has been recommended by current practice guidelines in case of inadequately sampled numbers of lymph nodes, even if no involvement of lymph nodes by tumor cells is found [12]. However, data are conflicting, and not all studies showed improved outcome for CRC patients when more lymph nodes had been retrieved [13–15]. The number of retrieved lymph nodes may depend on different factors, including surgical radicality and dedicated pathological work-up, but also on patient- and tumor-specific factors [6, 7].

International guidelines currently recommend sampling of a minimum number of 12 lymph nodes. Evidence, however, is weak, and it is not known, whether the sampling of more than 12 nodes improves staging accuracy and prognosis [6, 7]. It is therefore an ongoing debate how many lymph nodes need to be sampled for optimal staging. It is likewise unclear whether the cut-off value of 12 nodes should be adjusted by patient- or tumor-specific factors.

Therefore, our study aimed to analyze different clinicopathological factors that determine the extent of lymph node retrieval and to evaluate the prognostic impact of the extent of lymph node retrieval in a large cohort of CRC patients.

## Patients and methods

### Patient cohort

Our cohort consisted of 381 patients including 166 (44%) females and 215 (56%) males (ratio 1:1.3). Mean age was 68.5 years (median 70.1, range 28–93). Case selection of our cohort has been described in detail previously [16]. Briefly, 400 CRC patients treated from January 1992 through December 2000 at one institution (Medical University of Graz, Austria) were randomly selected from the CRC database of the Institute of Pathology, Medical University of Graz. We excluded patients with T1 cancer treated by endoscopic polypectomy, patients that received neoadjuvant chemotherapy or radiotherapy, and patients with syn- or metachronous invasive cancers originating from the colorectum or other sites. In total, 381 resection specimens from 400 patients (95%) were available for review pathology. AJCC/UICC stage determined treatment of our patients. Stage III patients received adjuvant chemotherapy with 5-fluorouracil/folinic acid according to the Mayo Clinic regimen [17]. Stage I and II patients did not receive adjuvant therapy. Patients with stage IV disease were treated with different combination chemotherapy regimens according to the choice of the treating physician. Follow-up of our cohort has been described in a previous publication [16]. It included at least chest X-rays and abdominal ultrasound every 6 months for the first 3 years and yearly thereafter. Disease progression was defined as local tumor recurrence or development of distant metastasis.

The investigation was carried out in accordance with the Declaration of Helsinki. The study was approved by the Institutional Review Board of the Medical University of Graz, Austria. For this retrospective study, formal consent of each patient is not required.

### Pathological evaluation

Original histopathological slides were independently re-evaluated by two gastrointestinal pathologists (M.J.P. and C.L.). Tumor stage was assessed according to the 7th edition of the AJCC/UICC TNM classification [4]. Histological tumor type and tumor grade were analyzed according to the WHO guidelines [18]. Tumors located in the rectum or at the rectosigmoid junction were summarized as rectal cancers. Tumors originating from sigmoid colon to the left colonic flexure were defined as left-sided cancers, while tumors located from transverse colon to caecum were defined as right-sided cancers. The presence of lymph and/or blood vessel invasion was assessed as carcinoma being present in vessels with an unequivocal endothelial lining (lymphatic invasion) or in vessels with a thick vascular wall and red blood cells in the lumen (blood vessel invasion) [16]. Mismatch-repair (MMR) status was assessed by immunohistochemistry as described earlier, using antibodies directed against MLH1, MSH2, and MSH6 [19]. The loss of immunoreactivity for at least one of the three markers characterized MMR-deficient tumors [20]. No special fat clearance or staining techniques were used for lymph node harvesting.

### Statistical analysis

Associations with AJCC/UICC stage, T-classification, N-classification, grade, MMR status, location, tumor size, lymphatic invasion, and venous invasion were analyzed using the Chi square test or Student’s *t* test. Coefficients of correlation were determined by Pearson’s *r*. Binary logistic regression model including an intercept was applied to assess the influence of primary tumor characteristics on the number of retrieved lymph nodes. The Hosmer-Lemeshow-test was used to assess goodness of fit of the model. Cause of death was determined by treating physicians and/or by chart review and was corroborated by death certificates if available. Progression-free survival (PFS) and cancer-specific survival (CSS) were investigated using the Kaplan-Meier method and compared by the log-rank test. For multivariable testing, Cox proportional hazards regression models were performed. Statistical calculations were performed using SPSS version 20.0 (IBM, Armonk, NY, USA). All reported *p* values were two-sided with significance at *p* < 0.05.

## Results

**Fig. 1:**
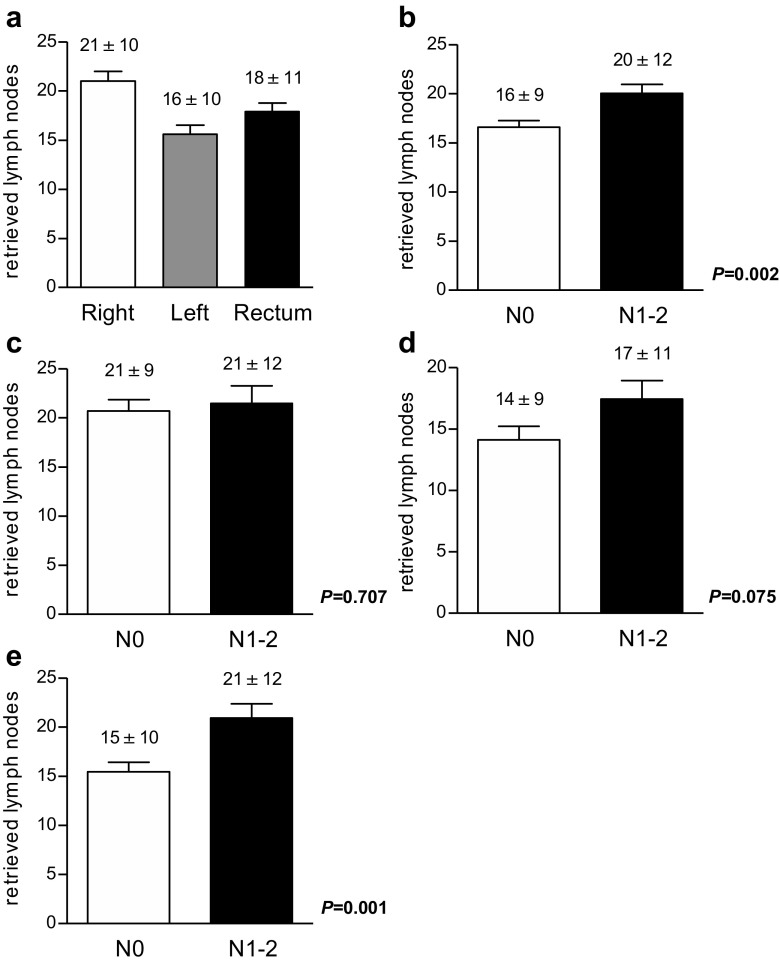
Numbers of retrieved lymph nodes in right-sided colon cancers, left-sided colon cancers, and rectum cancers (**a**). Numbers of retrieved lymph nodes in patients with node negative and node positive tumors analyzing all cases (**b**). Restricted to right-sided tumors (**c**). Restricted to left-sided tumors (**d**). Restricted to rectum cancers (**e**)

A mean number of 18.1 ± 10.7 lymph nodes (median 16, range 1–66) were retrieved in our patients. Specifically, the number was 21 ± 10.5 (median 20, range 1–65) in right-sided, 15.6 ± 9.8 (13, 1–66) in left-sided, and 17.9 ± 11 (15, 3–64) in rectal tumors (Fig. 1a). Twelve or more lymph nodes were sampled in 270 (70.9%) cases. Positive lymph nodes were detected in 168 (44%) patients. The number of retrieved lymph nodes was higher in patients with lymph node positive tumors compared with patients with lymph node negative tumors (mean 20 ± 11.8 versus 16.6 ± 9.5; *p* = 0.002, Fig. 1b). This difference was particularly pronounced in patients with rectal cancer (N0 15.4 ± 9.3 retrieved lymph nodes versus N1/2 20.9 ± 12.3, *p* = 0.001) and almost significant in left-sided colon cancers (N0 14.1 ± 8.9 versus N1/2 17.5 ± 10.6, *p* = 0.075), while no difference was observed in right-sided cancers (N0 20.7 ± 9.1 versus N1/2 21.5 ± 12.1, *p* = 0.707, Fig. 1c–e).

We categorized the number of retrieved lymph nodes into four groups (1–9, 10–19, 20–29, and >29). With this classification, a higher number of sampled nodes was significantly associated with higher T-classification (*p* < 0.001), higher AJCC/UICC stage (*p* < 0.001), larger tumor size (*p* = 0.030), and right-sided tumor location (*p* < 0.001). Association with N-classification was nearly significant (*p* = 0.059). There was no association with age, sex, grade, mismatch-repair status, and lymph or blood vessel invasion (Table 1).

**Table 1:** Associations of the number of retrieved lymph nodes with different clinicopathological parameters

		1–9 Lymph nodes		10–19 Lymph nodes		20–29 Lymph nodes		>29 Lymph nodes		*p* value
Age	≤70	40	(21.1%)	77	(40.5%)	51	(26.8%)	22	(11.6%)	0.95
	>70	39	(20.4%)	83	(43.5%)	48	(25.1%)	21	(11%)	
Sex	Female	40	(18.6%)	96	(44.7%)	56	(26%)	23	(10.7%)	0.56
	Male	39	(23.5%)	64	(38.6%)	43	(25.9%)	20	(12%)	
T-classification	1	12	(42.9%)	13	(46.4%)	2	(7.1%)	1	(3.6%)	<0.001
	2	23	(32.9%)	39	(55.7%)	5	(7.1%)	3	(4.3%)	
	3	37	(17%)	82	(37.6%)	74	(33.9%)	25	(11.5%)	
	4	7	(10.8%)	26	(40%)	18	(27.7%)	14	(21.5%)	
N-classification	0	51	(23.9%)	94	(44.1%)	49	(23%)	19	(8.9%)	0.059
	1	19	(22.9%)	28	(33.7%)	26	(31.3%)	10	(12%)	
	2	9	(10.6%)	38	(44.7%)	24	(28.2%)	14	(16.5%)	
AJCC/UICC stage	I	27	(33.3%)	45	(55.6%)	6	(7.4%)	3	(3.7%)	<0.001
	II	18	(15%)	47	(39.2%)	40	(33.3%)	15	(12.5%)	
	III	20	(15.9%)	50	(39.7%)	38	(30.2%)	18	(14.3%)	
	IV	14	(25.9%)	18	(33.3%)	15	(27.8%)	7	(13%)	
Tumor grade	1	35	(23.5%)	58	(38.9%)	43	(28.9%)	13	(8.7%)	0.27
	2	29	(20%)	66	(45.5%)	36	(24.8%)	14	(9.7%)	
	3	10	(16.4%)	28	(45.9%)	11	(18%)	12	(19.7%)	
	4	5	(19.2%)	8	(30.8%)	9	(34.6%)	4	(15.4%)	
L	L0	50	(19.6%)	112	(43.9%)	69	(27.1%)	24	(9.4%)	0.27
	L1	29	(23%)	48	(38.1%)	30	(23.8%)	19	(15.1%)	
V	V0	62	(21.1%)	124	(42.2%)	77	(26.2%)	31	(10.5%)	0.87
	V1	17	(19.5%)	36	(41.4%)	22	(25.3%)	12	(13.8%)	
Tumor size	≤4.5	28	(30.8%)	38	(41.8%)	18	(19.8%)	7	(7.7%)	0.030
	>4.5	51	(17.6%)	122	(42.1%)	81	(27.9%)	36	(12.4%)	
Tumor location	Right	13	(12.1%)	34	(31.8%)	43	(40.2%)	17	(15.9%)	<0.001
	Left	35	(31.8%)	44	(40%)	23	(20.9%)	8	(7.3%)	
	Rectum	31	(18.9%)	82	(50%)	33	(20.1%)	18	(11%)	
MMR status	Deficient	5	(21.7%)	10	(43.5%)	6	(26.1%)	2	(8.7%)	0.95
	Proficient	72	(20.6%)	147	(42%)	90	(25.7%)	41	(11.7%)	

In a binary logistic regression model, tumor size, T-classification, N-classification, and right tumor localization proved to be to independently associated with the retrieval of more than the recommended 12 lymph nodes (Table 2).

**Table 2:** Binary logistic regression analysis of factors predicting retrieval of ≥12 lymph nodes in CRC

Variable	Level	HR	CI	*p* value
Tumor size	>4.5	2.12	1.26–3.56	0.005
T-classification	>2	2.87	1.70–4.85	<0.001
N-classification	Positive	1.89	1.14–3.13	0.014
Location	Right	2.04	1.18–3.57	0.012

*HR* hazard ratio, *CI* confidence interval

**Fig. 2:**
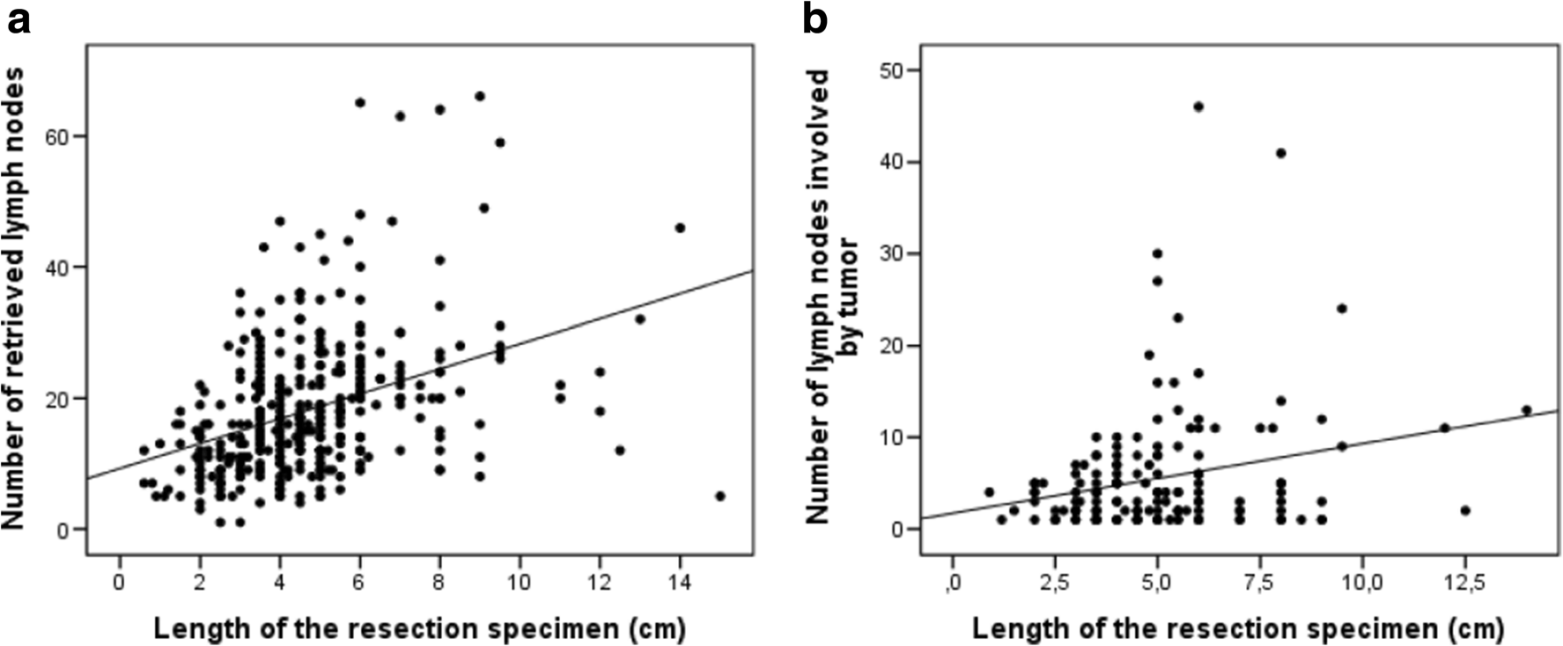
Length of the resection specimen related to the number of retrieved lymph nodes (**a**). The number of lymph nodes involved by tumor (**b**)

Of note, the length of the surgical resection CRC specimen (data available for 359 patients) correlated positively with the number of retrieved lymph nodes (*r* = 0.436; *p* < 0.001) and with the number of lymph nodes involved by tumor cells (*r* = 0.18; *p* = 0.001). That is, the longer the CRC specimen, the higher was the number of retrieved and also the number of positive lymph nodes (Fig. 2).

### Survival analysis

Analyzing 350 out of 381 (92%) patients with available follow-up data, we observed progressive disease in 141 (40%) patients after a mean (median) follow-up of 56 (45) months (range 1–182). Mean time to progression was 15 months (median 7, range 0–88). At the end of follow-up, 118 (34%) patients had died from cancer [16].

Disease progression occurred in 42.5, 36.1, 40, and 47.5% of patients with 1–9, 10–19, 20–29, and >29 sampled lymph nodes (*p* = 0.684), respectively. In addition, 34.3, 32.2, 33.0, and 37.5% of patients with 1–9, 10–19, 20–29, or >29 retrieved lymph nodes died of disease (*p* = 0.885).

When we analyzed our cohort using the cut-off of 12 lymph nodes recommended by the AJCC/UICC TNM staging system, we observed disease progression in 41.2% of patients with ≤12 compared with 39% of patients with >12 retrieved lymph nodes (*p* = 0.563). In addition, 32.8% of patients with ≤12 and 33.8% of patients with >12 retrieved lymph nodes died of disease (*p* = 0.879).

**Fig. 3:**
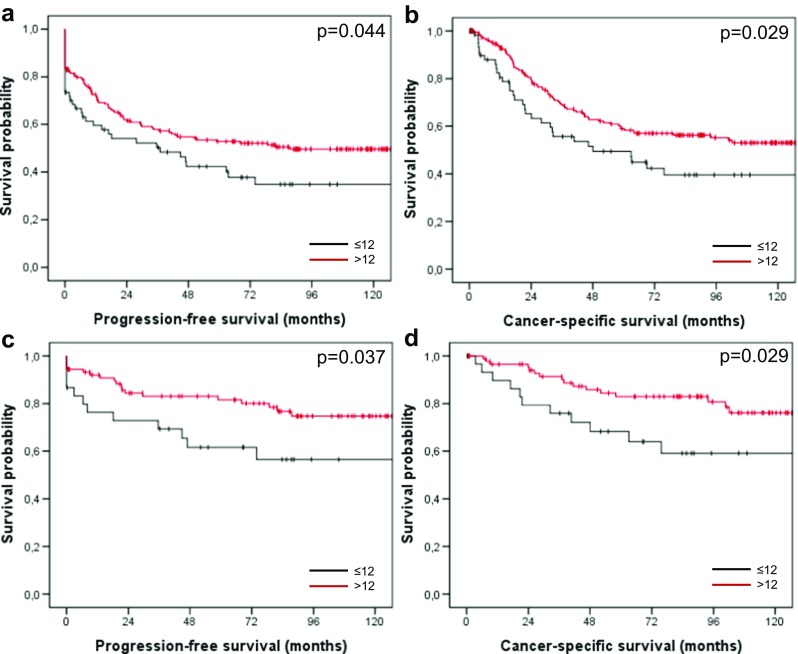
Progression-free (**a**) and cancer-specific survival (**b**) of patients with T3/T4 cancer related to the number of retrieved lymph nodes (≤12 versus >12 nodes); progression-free (**c**) and cancer-specific survival (**d**) of patients with T3/T4 N0 cancer related to the number of retrieved lymph nodes (≤12 versus >12 nodes)

When we restricted analysis to patients with T3 or T4 tumors irrespective of nodal status (*n* = 283), we observed disease progression in 57.8% of patients with ≤12 compared with 46.6% of patients with >12 retrieved lymph nodes (*p* = 0.044; Fig. 3a). Actuarial 5-year PFS rates were 42 and 52%, respectively. In addition, 48.4% of patients with ≤12 and 39.8% of patients with >12 retrieved lymph nodes died of disease (*p* = 0.029; Fig. 3b). Actuarial 5-year CSS rates were 52 and 61%, respectively.

In patients with T3/T4, N0 disease (*n* = 130), disease progression occurred in 40% of patients with ≤12 compared with 21.6% of patients with >12 retrieved lymph nodes (*p* = 0.037; Fig. 3c). Actuarial 5-year PFS rates were 60 and 81%, respectively. In addition, 36.6% of patients with ≤12 and 18.2% of patients with >12 retrieved lymph nodes died of disease (*p* = 0.029; Fig. 3d). Actuarial 5-year CSS rates were 67 and 84%, respectively.

In Cox proportional hazards regression analysis, lymph node retrieval prove to be an independent prognostic factor for PFS and CSS of patients with T3/T4 tumors irrespective of nodal involvement (Table 3). When we performed Cox analysis in T3/T4 N0 patients, again lymph node retrieval proved to be an independent prognostic variable for PFS (HR = 0.48, 95% CI = 0.23–0.99, *p* = 0.046) and OS (HR = 0.41, 95% CI = 0.19–0.90, *p* = 0.026), while age >70 years, female gender and grading did not show independent prognostic impact for both PFS (*p* = 0.84, *p* = 0.82, and *p* = 0.75, respectively) or OS (*p* = 0.47, *p* = 0.95, and *p* = 0.51, respectively).

**Table 3:** Multivariable analysis of prognostic factors for PFS and CSS in T3/T4 CRCs

		PFS			CSS		
Variable	Level	HR	CI	*p* value	HR	CI	*p* value
Age	>70	1.15	0.80–1.63	0.45	1.51	1.03–2.20	0.036
Gender	Female	1.13	0.79–1.62	0.51	1.18	0.80–1.74	0.41
Grade	>2	1.24	0.86–1.77	0.25	1.75	1.19–2.58	0.004
N-retrieval	>12	0.63	0.43–0.95	0.025	0.54	0.35–0.82	0.004
N-classification	>0	4.32	4.31–2.84	<0.001	4.61	2.95–7.21	<0.001

*PFS* progression-free survival, *CSS* cancer-specific-survival, *HR* hazard ratio, *CI* confidence interval, *N-retrieval* lymph node retrieval

## Discussion

The prognosis of patients with CRC after tumor resection depends substantially on the presence of lymph nodes involved by tumor cells. The number of sampled (that is the number of histologically analyzed) lymph nodes has therefore been discussed as marker for adequate staging, quality of surgical therapy and pathological work-up, but also as independent prognostic marker guiding therapeutic decisions [6, 7].

According to our data, retrieval of more than 12 lymph nodes proved to be a significant prognostic factor in locally advanced (T3/T4) CRC in both univariable and multivariable analyses. Moreover, high T-classification and tumor stage, tumor size and location, as well as length of the resection specimen were all significantly associated with lymph node count. In concordance with our data, a systematic review [8] and further recent studies [9, 21–24] demonstrated significant prognostic impact of lymph node count in stage II disease. A reason for this may be the better selection of patients who are truly node negative and are thus cured by surgery alone [7]. However, this view has been challenged by publications arguing that a higher number of retrieved lymph nodes did not increase the proportion of stage III disease [25, 26]. Also, in stage III cases and in mixed populations, several studies showed significant prognostic impact [8–11, 27, 28]. A more thorough clearance of tumor cells by improved lymphadenectomy may explain the observed prognostic influence [7]. Accordingly, studies including ours, have demonstrated an association of lymph node retrieval with the length of the examined operation specimen [13, 24, 29–31].

However, independent of surgical extent und thoroughness of the pathological work-up, tumor- and/or patient-specific factors are influencing lymph node retrieval in colorectal cancer. According to our data, a higher number of lymph nodes was harvested in patients with lymph node involvement by tumor. This association has been reported previously [11, 21, 23]; however, not all studies showed this association [10]. Furthermore, T-classification and higher tumor stage were significantly associated with higher lymph node count. Both associations are in the line with literature data showing associations of lymph node retrieval with T-classification [13, 21, 23, 32] and AJCC/UICC stage [11, 13, 14, 21, 32–35]. Also, tumor size is, according to previous studies, an established predictor of lymph node yield [11, 13, 23, 29, 31, 36] that could be confirmed by our study. Other previously described factors were not found to be related to lymph node yield in our study. For instance, younger patient age has repeatedly been reported to be significantly associated with lymph node count [9, 11, 13, 14, 23, 29, 31–33, 35, 37], but not in our cohort.

Another interesting determining factor for lymph node yield is tumor location. Apart from our analysis, several other studies have demonstrated higher numbers of lymph nodes retrieved from patients with right-sided, compared with left-sided cancers [9, 13, 29, 32, 34–38]. Differences in embryonic development or a greater length of the mesenteric root have been discussed as possible cause [7]. However, also a higher inflammatory response to right-sided tumors, which are often microsatellite instable, has been proposed and found in previous analyses [36, 39]. We were not able to prove this association, but the number of mismatch-repair deficient tumors was rather low in our cohort.

Since patient- and tumor-related factors play a major role for lymph node retrieval, the value of efforts, which aim to increase the lymph node yield, has been challenged [40]. Also, since several studies failed to prove prognostic impact [13–15, 34, 36, 40]. These negative results may be due to different methodologies and heterogeneous study populations including different stages, both colon and rectum cancers, different cut-off values, but also small sample sizes. Of note, median numbers of retrieved lymph nodes vary greatly among published reports, according to a review by McDonald et al. [6] ranging from 6 to 21.

It has to be mentioned as limitation that our study cohort is heterogeneous with respect to inclusion of both, colon and rectum cancers. We excluded patients with rectal cancer that had undergone neoadjuvant chemotherapy or radiotherapy before surgery to keep our study cohort more homogenous. Of note, previous studies have revealed lower numbers of retrieved lymph nodes after neoadjuvant treatment [21, 23, 33, 41]. Another limitation of our study is the retrospective character of our analysis. Previous studies have shown that factors related to surgical procedure were related to lymph node harvest, including open versus laparoscopic approach. Those factors could not be analyzed here in detail as data were not available in this regard. Of note, with complete mesocolic excision (CME), a standardized surgical procedure is now available that leads to better surgical specimens and allows improved quality control. Therefore, future studies dealing with lymph node yield in colon cancer surgery should be done with pathologically graded CME specimens to increase comparability [42].

In conclusion, higher AJCC/UICC stage, tumor size, and right-sided location, as well as the length of the operation specimen were factors associated with lymph node retrieval. Rational cut-off values defining the minimum number of lymph nodes to be assessed for staging of CRC may thus be adjusted by these variables. According to our data, analysis of more than 12 lymph nodes is significantly associated with improved outcome in patients with locally advanced (T3/T4) disease.
